# Unexplained Recurrent Pregnancy Loss and Emerging Endometrial Biology: A Clinically Oriented Narrative Review

**DOI:** 10.7759/cureus.114105

**Published:** 2026-08-07

**Authors:** Daichi Inoue

**Affiliations:** 1 Department of Reproductive Medicine, Nishitan ART Clinic Sakae, Nagoya, JPN

**Keywords:** chronic endometritis, endometrial immunity, endometrial microbiome, narrative review, recurrent pregnancy loss, reproductive failure

## Abstract

A substantial proportion of couples who experience recurrent pregnancy loss (RPL) complete a full guideline-based evaluation without an identifiable cause and are given a diagnosis of unexplained RPL. This category should be interpreted as the current limitation of clinically validated biological explanation rather than as the absence of underlying pathology, and it is within this diagnostic gap that interest in emerging endometrial biology has grown.

Recent advances in endometrial biology have generated substantial interest in three closely related investigational domains: the endometrial microbiome, chronic endometritis (CE), and the endometrial immune milieu. Each domain has independently been associated with reproductive failure in observational research, and growing evidence suggests potential biological interactions among them. However, significant methodological heterogeneity, absence of standardized diagnostic criteria, and insufficient prospective validation currently preclude routine clinical implementation.

This narrative review synthesizes current evidence in each of these domains and weighs their relevance to how unexplained RPL is managed. The review concludes that abnormalities involving endometrial microbial composition, chronic endometrial inflammation, and local immune dysregulation represent biologically plausible contributors to unexplained pregnancy loss in selected women, but that the principal barriers to clinical adoption are reproducibility, methodological standardization, and demonstrated clinical utility rather than biological plausibility alone. Major professional guidelines currently advise against routine use of these tests outside a research context. Emerging endometrial biology should therefore be interpreted as a framework for ongoing scientific inquiry, not as a basis for expanded clinical testing. Incorporation into routine practice will require standardized methodology, independent multicenter validation, and prospective demonstration that diagnosis and treatment improve clinically meaningful reproductive outcomes.

## Introduction and background

Recurrent pregnancy loss (RPL) is generally defined as the loss of two or more pregnancies and, depending on the definition applied, is estimated to affect roughly one to two in every hundred reproductive-age couples [1,2]. It constitutes one of the most challenging disorders encountered in reproductive medicine, combining significant reproductive failure with profound psychological consequences for affected individuals and couples. It should be noted that the definition of RPL is not uniform across authorities: the number of losses required (two versus three) and whether biochemical or other non-visualized pregnancy losses are counted differ between the European Society of Human Reproduction and Embryology (ESHRE) and other bodies [3]. In this review, RPL is used broadly and, unless otherwise specified, in accordance with the ESHRE definition, which counts non-visualized pregnancy losses [3]. Current international guidelines recommend systematic evaluation for established causes, including parental chromosomal abnormalities, uterine structural anomalies, antiphospholipid syndrome, selected endocrine disorders, and, where available, genetic analysis of products of conception (POC) [4,5]. Despite the availability of these investigations, a large share of couples - in most cohort series between a third and a half - still complete this recommended work-up without an identifiable explanation.

Unexplained RPL should not be interpreted as evidence that no biological abnormality exists [4,5]. Rather, it reflects the current limitations of clinically validated explanation. Successful pregnancy depends upon coordinated interaction between embryo quality and maternal endometrial function across multiple biological dimensions. While embryonic chromosomal abnormalities account for a large proportion of sporadic first-trimester miscarriages, they do not explain all recurrent losses, and embryonic and maternal factors should not be viewed as mutually exclusive pathways [2].

Recent advances in molecular biology and next-generation sequencing have stimulated growing interest in three interrelated aspects of endometrial biology - its microbial communities, the entity of chronic endometritis (CE), and its local immune environment [6,7]. Each has been linked, in observational work, to reproductive failure, and there are early indications that these processes interact in ways that may be relevant to the broader phenomenon of unexplained pregnancy loss [8,9]. However, current evidence in each domain remains substantially limited by methodological heterogeneity, lack of standardized definitions and diagnostic thresholds, and insufficient prospective validation in women with RPL as the primary outcome [10,11]. For this reason, major guidelines presently confine such testing to research or highly selected clinical situations rather than routine care.

The objective of this narrative review is not to advocate expansion of clinical testing but to help clinicians interpret emerging biological evidence within the framework of current evidence-based practice. The review weighs the strengths and limitations of the evidence in each of the three areas and considers how these findings should inform the clinical management of unexplained RPL.

Although each of these three domains has been reviewed individually, they are almost always considered in isolation. The novelty of this review lies in evaluating all three against a single, explicit yardstick - biological plausibility, methodological limitations, evidence that treatment improves live birth, and the current position of major guidelines - so that they can be compared directly rather than domain by domain. This unified appraisal allows a clinically actionable message to emerge: that the three fields, despite differing biology, share the same evidentiary gap and therefore call for the same cautious response when patients ask about testing and treatment.

Scope and evidence base

This narrative review was undertaken to provide a clinically oriented synthesis of current evidence regarding emerging endometrial biology in unexplained RPL. The review focuses on three investigational domains that have attracted increasing attention in reproductive medicine and that are already being offered to patients as commercial tests or empirical treatments: endometrial microbial composition, CE, and local endometrial immune function. A structured literature search was performed using PubMed/MEDLINE, with the most recent search executed in July 2026. The principal search terms included recurrent pregnancy loss, recurrent miscarriage, endometrial microbiome, uterine microbiome, chronic endometritis, endometrial immune, implantation, and pregnancy loss. Additional publications were identified through manual review of reference lists from eligible articles and contemporary international clinical practice guidelines.

Because the objective of this article was to provide a clinically relevant narrative synthesis rather than a quantitative evidence aggregation, studies were selected according to their contribution to current clinical understanding, with particular emphasis on distinguishing established evidence that supports guideline-based practice from emerging biological observations that remain investigational. Priority was given to international guidelines, systematic reviews, meta-analyses, prospective cohort studies, randomized controlled trials, and high-quality narrative reviews published primarily between January 2000 and the search date, while experimental studies were included when they provided mechanistic insight directly relevant to implantation or early pregnancy maintenance. Throughout the review, evidence was interpreted in the context of current recommendations from the ESHRE, the American Society for Reproductive Medicine (ASRM), and the 2025 Japanese recommendations for the management of recurrent pregnancy loss [3-5]. To allow the three domains to be compared on equal terms rather than in isolation, each was appraised against the same four criteria: biological plausibility, methodological limitations, evidence that treatment improves live birth, and the current position of major guidelines. This unified appraisal is summarized in Table 1.

**Table 1:** Biological rationale, methodological limitations, evidence for treatment benefit, and current guideline position across the three domains of emerging endometrial biology in unexplained recurrent pregnancy loss. RPL: recurrent pregnancy loss

Domain	Biological rationale	Principal methodological limitations	Evidence that treatment improves live birth	Current guideline position
Endometrial microbiome	Lactobacillus-dominant communities are associated with a more favorable peri-implantation environment; a dysbiotic microbiome is proposed to promote a pro-inflammatory environment incompatible with implantation.	Very low microbial biomass with high susceptibility to contamination; transcervical sampling prone to cross-contamination from the vagina and cervix; no standardization of sampling, DNA extraction, sequencing platform, 16S rRNA variable region, or bioinformatic pipeline; no accepted reference definition of a normal endometrial microbiome.	Not demonstrated. Normalization of microbial composition by antibiotics, probiotics, or other interventions has not been shown to improve live-birth rates in adequately powered prospective trials.	Routine endometrial microbiome testing is not recommended.
Chronic endometritis	Chronic endometrial inflammation may disrupt endometrial receptivity and decidualization; interleukin-1, interleukin-6, and tumor necrosis factor-alpha may impair trophoblast invasion.	Plasma-cell threshold for diagnosis ranges from one to five or more per high-power field; variation in biopsy site, cycle timing, CD138 staining protocol and interpretation, and interobserver agreement; reported prevalence in RPL ranges from fewer than 10% to more than 50%.	Not confirmed. A multicenter, double-blind, placebo-controlled randomized trial of doxycycline found no increase in live-birth rate (relative risk: 1.02, 95% credible interval: 0.85-1.21).	Universal screening in unexplained RPL is not recommended.
Endometrial immune milieu	Implantation requires regulated maternal immune adaptation; disruption of uterine natural killer cells, regulatory T cells, and cytokine balance may impair implantation or early placental development.	Peripheral blood parameters may not reflect the endometrial compartment; marked variations in menstrual cycle phase, hormonal stimulation, and laboratory methods; no accepted reference standards, analytical harmonization, or thresholds defining clinically meaningful abnormality.	Not demonstrated. Corticosteroids, intravenous immunoglobulin, intralipid, and other immunomodulatory therapies have not shown consistent benefit and carry cost and risk.	Routine immune testing and empirical immune therapy are recommended against.

## Review

Unexplained RPL after guideline-based evaluation

Current international guidelines recommend systematic evaluation for established causes of RPL, including parental karyotyping to detect balanced chromosomal rearrangements, imaging assessment of uterine anatomy, serological testing for antiphospholipid antibodies, thyroid function testing, and, where available, chromosomal analysis of miscarriage tissue to determine the genetic basis of individual losses [3-5,12]. These investigations have substantially improved clinical management by identifying conditions for which evidence-based interventions exist: antiphospholipid syndrome, for example, is now routinely treated with aspirin and heparin with demonstrable improvement in live-birth rates, and congenital uterine anomalies amenable to surgical correction can be identified and addressed [1,2].

Despite this progress, a substantial proportion of couples - conservatively estimated at one-third to one-half in most contemporary cohort studies - complete recommended evaluation without an identifiable cause [2]. This persistent diagnostic category reflects not inadequacy in routine clinical care but the genuine limitations of currently validated clinical investigations. Successful implantation and early pregnancy maintenance depend upon numerous local biological processes occurring within the endometrium during the peri-implantation window, many of which remain measurable only within research settings and have not yet been validated as clinically actionable biomarkers.

The clinical weight of unexplained RPL is not captured by prevalence figures alone. Recurrent loss is associated with substantial psychological morbidity, including anxiety, depression, and grief that may persist into and beyond a subsequent pregnancy, and the absence of an identifiable cause can intensify distress by denying couples an explanation and a clear target for treatment [2]. This diagnostic vacuum is precisely what makes emerging biological explanations so appealing to patients and clinicians alike, and it helps to explain why microbiome, CE, and immune testing have diffused into practice ahead of the evidence required to justify them. A central task of the clinician is therefore to hold two messages simultaneously: that the absence of a validated explanation does not mean the absence of biology, and that the presence of a plausible biological hypothesis does not by itself justify testing or treatment.

It is equally important to place the prognosis of unexplained RPL in perspective. Even after two or more losses, and in the absence of treatable factors, the probability of a subsequent live birth remains substantial for many couples, particularly younger women without additional risk factors [2]. This favorable natural history has two consequences for the interpretation of the evidence that follows. First, uncontrolled studies of any intervention will tend to report apparent success simply because many untreated couples would have conceived successfully regardless; this is a recurring source of bias in the microbiome, CE, and immune literature. Second, the bar for adopting a new test or treatment should be correspondingly high, because interventions must improve on an already reasonable baseline rather than rescue an otherwise hopeless situation.

The diagnosis of unexplained RPL should therefore be viewed as a dynamic rather than static category. It does not signify the absence of any underlying biological abnormality, but rather the current boundary of validated clinical explanation. Because pregnancy depends on the coordinated interaction of embryonic and maternal factors, and because embryonic chromosomal abnormalities account for a large proportion of sporadic first-trimester losses but do not explain all recurrent losses, embryonic and maternal contributions should be regarded as complementary rather than mutually exclusive pathways [12]. It is against this background - a large, biologically heterogeneous group of couples seeking an explanation - that interest in the endometrial microbiome, CE, and the endometrial immune milieu has grown, and it is within this clinical context that the strength of the supporting evidence must be judged. A full account of the genetic contribution to RPL - including parental balanced translocations, embryonic aneuploidy, and the genetic testing of POC - is beyond the scope of this review and has been covered in detail elsewhere [1,2,12]; here, chromosomal factors are treated as an established diagnostic category, and the focus is deliberately placed on the endometrial microenvironment that may remain relevant once such causes have been excluded.

Endometrial microbiome

From the Sterile-Womb Hypothesis to a Resident Endometrial Microbiota

The traditional concept of a sterile uterine cavity has been fundamentally revised over the past decade by advances in culture-independent sequencing technologies, most notably next-generation sequencing of the 16S ribosomal RNA (rRNA) gene [13]. Studies applying these techniques have reported the presence of microbial communities within the endometrium, and several observational investigations have described differences in endometrial microbial composition between women with reproductive failure and fertile controls [14,15]. In particular, reduced relative abundance of *Lactobacillus* species - which constitute the dominant organisms in the healthy lower female reproductive tract - and increased microbial diversity have been associated with infertility, recurrent implantation failure, and RPL in some studies [16,17]. It should be emphasized, however, that the endometrial niche differs fundamentally from the vaginal niche in which *Lactobacillus* dominance was first characterized: the absolute microbial load is orders of magnitude lower, the community is far less stable across the menstrual cycle, and whether an endometrium-specific *Lactobacillus*-dominant “healthy” state can be defined at all remains an open question. Reports of an endometrial microbiome should therefore be read as evidence that microbial DNA can be detected within the cavity, not as proof of a stable, functionally characterized resident community comparable to that of the gut or vagina.

Biological Rationale for a Role in Implantation

The biological rationale for endometrial microbiome involvement in implantation is nonetheless plausible [18]. *Lactobacillus*-dominant communities have been associated with a more favorable peri-implantation endometrial environment, potentially through maintenance of epithelial barrier integrity, production of bacteriocins and organic acids that suppress pathogenic overgrowth, and modulation of local inflammatory signaling toward a state compatible with embryo apposition and invasion [19,20]. Conversely, a non-*Lactobacillus*-dominant or dysbiotic endometrial microbiome has been proposed to promote a pro-inflammatory microenvironment that is incompatible with normal implantation, for example by increasing local production of inflammatory cytokines and shifting the balance of resident immune populations [10]. These mechanistic pathways are conceptually coherent and are consistent with what is known about host-microbe interactions at other mucosal surfaces, but they remain hypothetical in the specific context of human reproductive outcomes, having been demonstrated largely in association studies, in vitro models, or by analogy rather than through interventional human data [21]. The plausibility of the mechanism should not be mistaken for evidence that it operates as a clinically important cause of pregnancy loss.

Methodological Limitations

The principal limitation of current endometrial microbiome research is not a shortage of biological hypotheses but the absence of methodological standardization [22]. The endometrium contains extremely low microbial biomass, which makes contamination during specimen collection, DNA extraction, and sequencing library preparation a major and underappreciated concern. In low-biomass samples, reagent- and laboratory-derived sequences can rival or exceed the true biological signal, so that apparent microbiome differences may in part reflect differences in background contamination rather than in endometrial biology [23,24]. Transcervical sampling is particularly prone to cross-contamination from the high-biomass vaginal and cervical compartments, which is frequently not controlled for, and studies vary in whether they attempt to distinguish a genuine endometrial signal from vaginal carryover [23,24]. Studies also differ considerably with regard to endometrial sampling technique (biopsy versus uterine lavage versus embryo-transfer catheter tip), DNA extraction protocol, sequencing platform, the specific 16S rRNA variable regions targeted, bioinformatic pipeline, and statistical approach. Because the choice of 16S variable region alone can substantially alter the apparent community composition, results are often not directly comparable between laboratories [25]. No universally accepted reference definition of a normal endometrial microbiome currently exists, which means that classifications such as “dysbiotic” or “*Lactobacillus*-depleted” are defined differently across studies [26]. Patient populations additionally vary substantially with respect to infertility diagnosis, previous pregnancy history, menstrual cycle phase, hormonal treatment, recent antibiotic exposure, and concurrent lower-tract microbial status. These sources of variation collectively reduce reproducibility across studies and limit the comparability of published findings.

Association Versus Causation, and the State of Interventional Evidence

A further limitation is the conflation of association with causation [6]. Although several studies have reported correlations between endometrial microbial profiles and reproductive outcomes, current evidence is insufficient to establish that dysbiosis directly causes pregnancy loss rather than being a coincident or downstream biological finding [27-30]. A dysbiotic profile could equally be a consequence of the same underlying endometrial dysfunction that leads to pregnancy loss, or a marker of a shared upstream factor, without lying on the causal pathway itself. Systematic reviews and meta-analyses of reproductive tract microbiota and miscarriage have reached cautious conclusions, constrained by precisely the heterogeneity described above [31,32]. Equally important, normalization of microbial composition - whether through antibiotic treatment, probiotic supplementation, or other interventions - has not yet been shown to consistently improve live-birth rates in adequately powered prospective trials, and much of the supporting data derives from assisted-reproduction populations rather than from women with unexplained RPL [33]. Accordingly, endometrial microbiome abnormalities should currently be interpreted as candidate biological associations rather than established causes of RPL, and microbiome profiles do not yet constitute actionable clinical biomarkers.

Current Guideline Position

Current international guidelines do not recommend routine endometrial microbiome testing in women with RPL [3-5]. This position reflects insufficient evidence for diagnostic reproducibility, clinical validity, and clinical utility, rather than a rejection of the underlying biological plausibility [34,35]. The distinction matters, because the absence of a recommendation is sometimes misread by patients and clinicians as a statement that the endometrial microbiome is irrelevant; the more accurate interpretation is that the tools available to measure it, and the evidence that acting on the result improves outcomes, have not yet reached the standard required for routine care.

A recurring conceptual difficulty in this literature is the relationship between the vaginal and endometrial compartments. Much of what is known about a “protective” *Lactobacillus*-dominant state derives from the vaginal microbiome, which is far more accessible and better characterized [19]. Several studies of RPL have accordingly examined the vaginal or cervicovaginal microbiome rather than the endometrium itself, reporting associations between reduced *Lactobacillus* dominance or increased diversity and pregnancy loss [28,29]. Whether these vaginal findings are valid surrogates for the endometrial environment, or whether they reflect a distinct compartment with its own relationship to reproductive outcome, remains unresolved. This distinction matters clinically, because a vaginal swab and an endometrial biopsy are very different propositions in terms of invasiveness, contamination risk, and interpretability, and conflating the two overstates how close the field is to a usable test.

Evidence from assisted-reproduction populations adds further nuance without resolving the central question. Systematic reviews of genital tract microbiota and assisted reproduction outcomes have reported associations between microbial composition and implantation or clinical pregnancy, but with the same heterogeneity and risk of confounding seen in the RPL literature, and with inconsistent definitions of a receptive microbial profile [32]. Studies examining whether a dysbiotic endometrium impairs blastocyst implantation in in-vitro fertilization patients illustrate both the promise and the limitations of this approach: associations are reported, but causal inference is constrained by study design, and extrapolation from assisted reproduction cohorts to spontaneously conceiving couples with unexplained RPL is not straightforward [33]. Taken together, the microbiome literature is best characterized as hypothesis-generating rather than practice-changing.

Chronic endometritis

Definition and Diagnosis

CE is characterized by persistent low-grade inflammation of the endometrial stroma and is histologically defined by the presence of plasma cells, most reliably identified using CD138 (syndecan-1) immunohistochemistry on endometrial biopsy specimens [36,37]. Unlike acute pelvic infection, CE is typically asymptomatic and therefore cannot be identified reliably on the basis of clinical symptoms or routine imaging; hysteroscopic findings, such as micropolyps and stromal edema, have been proposed as supportive signs but are neither sensitive nor specific enough to replace histology [38]. Several observational studies have reported a higher prevalence of CE among women with infertility, recurrent implantation failure, and RPL compared with fertile controls, and the condition has attracted considerable clinical interest precisely because, unlike many proposed contributors to reproductive failure, it appears potentially treatable with antibiotics [39].

Biological Rationale for a Role in RPL

The biological plausibility of CE as a contributor to RPL is supported by evidence that chronic endometrial inflammation may disrupt normal endometrial receptivity and the complex immunological adaptations required for successful implantation [7]. Inflammatory cytokines associated with CE - including interleukin-1, interleukin-6, and tumor necrosis factor-alpha - may impair the endometrial decidualization response and interfere with trophoblast invasion. Altered expression of adhesion molecules and aberrant B-cell infiltration have been proposed as additional mechanisms, together providing a mechanistic basis for recurrent early pregnancy loss [40]. There is also increasing interest in the interface between CE and the endometrial microbiome, with some data suggesting that specific microbial patterns accompany the histological picture of CE, although whether dysbiosis causes the inflammation, results from it, or merely coexists with it remains unresolved [41]. Demonstrating any of these mechanisms specifically in women with unexplained RPL, as opposed to infertility or implantation failure, remains an important evidence gap.

Diagnostic Reproducibility

The central challenge in the clinical evaluation of CE is not whether the condition exists, but whether it can be diagnosed reproducibly [38]. Published studies differ substantially with respect to the plasma-cell threshold required for diagnosis (ranging from one to five or more plasma cells per high-power field in various studies), the number of high-power fields examined, biopsy location within the uterine cavity, menstrual cycle timing at the time of biopsy, immunohistochemical staining protocol and interpretation, and interobserver agreement among pathologists [37]. Because CD138-positive plasma cells can be sparse and unevenly distributed, and because stromal cells at certain cycle phases may be misinterpreted, the same specimen can be classified differently by different observers or laboratories. As a consequence, patients classified as having CE according to one study’s diagnostic criteria would frequently not satisfy the criteria used by another, making it difficult to compare reported prevalence rates or to draw reliable conclusions about treatment effects across studies. Reported prevalence among RPL populations varies markedly across the literature, ranging from fewer than 10% to more than 50% depending on the thresholds and methods applied - a range so wide that it is itself an indicator of the underlying diagnostic instability [39].

Evidence for Treatment Benefit

Evidence for treatment efficacy is similarly limited. Several retrospective and observational studies have reported improved subsequent reproductive outcomes in women with CE who received antibiotic treatment, typically with doxycycline-based regimens directed at endometrial pathogens. Comparisons of “cured” versus “persistent” CE after treatment have suggested better outcomes in those whose histology normalized [40]. These findings generated substantial clinical interest, and antibiotic treatment of CE has been widely adopted in some fertility centers. However, the observational studies underlying these reports are predominantly retrospective, involve relatively small cohorts, and use heterogeneous diagnostic definitions and treatment protocols that limit generalizability. These are also susceptible to confounding, because women whose CE resolves may differ systematically from those in whom it persists. More recently, higher-quality prospective evidence has tempered this enthusiasm. A multicenter, double-blind, placebo-controlled randomized trial of doxycycline in women with recurrent miscarriage and CE (the CERM trial) was designed specifically to test whether antibiotic treatment increases the live-birth rate, and its protocol reflects the methodological rigor that the field has lacked [42]. Reported randomized data indicate no increase in live-birth rate with antibiotic treatment (relative risk approximately 1.02, 95% credible interval spanning unity), consistent with the view that the observational signal may have reflected confounding rather than a true treatment effect. Treatment efficacy must therefore currently be regarded as uncertain, and enthusiasm for empirical antibiotic treatment should be tempered accordingly [42].

Current Guideline Position

Current international guidelines do not recommend universal screening for CE in all women with unexplained RPL [3-5]. This recommendation reflects uncertainty regarding diagnostic reproducibility, treatment efficacy, and clinical utility, rather than evidence that CE lacks biological relevance to pregnancy outcomes. Standardized diagnostic criteria - including harmonized CD138 thresholds, biopsy timing, and reporting - and prospective randomized evidence linking CE diagnosis and treatment to improved live-birth outcomes are prerequisites for future guideline incorporation [38].

The relationship between CE and the endometrial microbiome deserves particular attention, because the two fields are increasingly discussed together and are sometimes marketed as a combined diagnostic package. Systematic evaluation of the endometrial microbiota in the pathogenesis of CE suggests that specific microbial patterns may accompany the histological picture of chronic inflammation, raising the possibility that CE represents, at least in part, a host response to a disturbed endometrial microbial community [41]. However, the direction of causation is unresolved: dysbiosis could drive inflammation, inflammation could reshape the microbial community, or both could be downstream of a shared upstream disturbance. Until this is clarified, the pragmatic implication is that combining two incompletely validated tests does not produce a validated one, and that a positive result on a bundled microbiome plus CE panel should not be over-interpreted.

The practical consequence of diagnostic instability is also worth making explicit for clinicians. Because the reported prevalence of CE in RPL spans an enormous range, a woman investigated at one center may be told she has a treatable cause of her losses, while the same biopsy interpreted elsewhere might be reported as normal. This variability not only undermines research comparability but can directly affect patient experience, generating either false reassurance or unwarranted treatment. Standardization of the CD138 threshold, the number of fields examined, the timing of biopsy within the cycle, and reporting terminology is therefore not merely an academic exercise but a prerequisite for equitable and reproducible clinical care [38].

Endometrial immune milieu

Immune Adaptation at the Maternal-Fetal Interface

Successful implantation requires a tightly regulated process of maternal immune adaptation rather than generalized immune suppression. The endometrium contains a highly specialized immune microenvironment composed of uterine natural killer (uNK) cells, macrophages, dendritic cells, regulatory T cells (Tregs), and a complex cytokine network that interacts continuously with endometrial stromal cells and the developing embryo [43]. Rather than mounting a rejection response against the semi-allogeneic conceptus, this system supports controlled trophoblast invasion, spiral artery remodeling, and establishment of maternal-fetal tolerance. Experimental and translational studies have established that disruption of this regulated immune balance - through excess pro-inflammatory signaling, deficiency of tolerogenic immune populations, or aberrant innate immune activation - may impair implantation or early placental development [11]. The conceptual attractiveness of this framework, together with the observation that RPL sometimes clusters with autoimmune phenomena, has made reproductive immunology one of the most intensively studied and most commercially exploited areas of the field.

Candidate Biomarkers and Why They Have Not Been Translated

This biological framework has stimulated decades of research into immune biomarkers in women with RPL [44]. Numerous candidates have been investigated, including peripheral NK cell number and cytotoxicity, uNK cell density on endometrial biopsy, circulating cytokine profiles, Th1/Th2 balance, Treg populations in peripheral blood and the endometrium, and HLA-related parameters including shared HLA-C alleles and killer immunoglobulin-like receptor (KIR) compatibility [45]. Despite this sustained investigative effort, none of these biomarkers has achieved the analytical validity, biological reproducibility, and clinical validation required for routine clinical use [11]. Several structural barriers explain this failure of translation. First, many published studies evaluate peripheral blood immune parameters - circulating NK cell activity or Treg frequency - that may not accurately reflect the local immune environment within the endometrium during the critical peri-implantation window, and the relationship between peripheral and uterine immune compartments is not straightforward, so that peripheral biomarkers may have limited predictive value for endometrial immune function [43,44]. Second, immune parameters fluctuate considerably according to menstrual cycle phase, hormonal stimulation, ongoing pregnancy, concurrent infection, and laboratory analytical method, so that in the absence of universally accepted reference standards and analytical harmonization, similar numerical results may not represent comparable biological states across laboratories or cohorts. Third, the biological significance of extreme values in either direction - and the thresholds that might define clinically meaningful abnormality - remains poorly established for most proposed biomarkers [11].

Gap Between Detection and Treatment

Perhaps the most important question is not whether immune abnormalities can be detected in women with RPL, but whether their detection leads to interventions that improve live-birth outcomes. To date, this question has not been answered affirmatively. Empirical immune therapies - including corticosteroids, intravenous immunoglobulin, intralipid infusion, low-molecular-weight heparin beyond thromboprophylactic indications, and other immunomodulatory interventions - have not consistently demonstrated sufficient benefit over standard care in women with unexplained RPL to justify routine clinical use, and trials have generally been small, heterogeneous, and inconsistent in their entry criteria. These interventions are not without consequence: they carry real costs and risks, including infection risk, thromboembolic risk, and financial and psychological burden, without established evidence of benefit. Current international guidelines consistently recommend against routine immune testing and empirical immune therapy in this population [3,4]. It bears emphasis that these recommendations reflect insufficient evidence for clinical utility - not rejection of the underlying biological concepts. The immune milieu at the maternal-fetal interface undoubtedly plays a central role in implantation and early pregnancy maintenance, and it remains an active and scientifically productive area of investigation [9,11,46]. The distinction, once again, is between a biological process being important and a biomarker or therapy being clinically useful.

The specific example of uNK cells illustrates the wider problem. uNK cells are the dominant leukocyte population in the peri-implantation endometrium and have a well-defined physiological role in spiral artery remodeling and trophoblast regulation, which makes them a biologically compelling candidate [43]. Yet efforts to translate uNK biology into a clinical test have foundered on precisely the barriers described above: peripheral blood NK measurements correlate poorly with uterine populations, endometrial uNK density varies with cycle timing and quantification method, and no threshold reliably separates women who will miscarry from those who will not. As a result, uNK testing has been offered commercially and used to justify immunosuppressive treatment without the validation that would be required of any other diagnostic assay, a situation that guideline bodies have explicitly cautioned against [11].

The therapeutic literature reflects the same tension between mechanism and evidence. Corticosteroids, intravenous immunoglobulin, and intralipid have each been advocated on the basis of plausible immunomodulatory mechanisms and encouraging uncontrolled series, but randomized evidence has been inconsistent and generally unsupportive of routine use, while exposing women to real risks and costs. Th1/Th2/Treg frameworks and HLA-C/KIR compatibility have similarly generated a large descriptive literature without yielding a validated, actionable test [45]. The pattern is by now familiar: a strong biological rationale, a proliferation of candidate markers and interventions, and a persistent failure to demonstrate that acting on any of them improves live birth. This is not an argument that immunology is irrelevant to implantation - it plainly is not - but rather that biological importance has not yet been converted into clinical utility.

Integrative interpretation: three domains, one unresolved question

**Figure 1:**
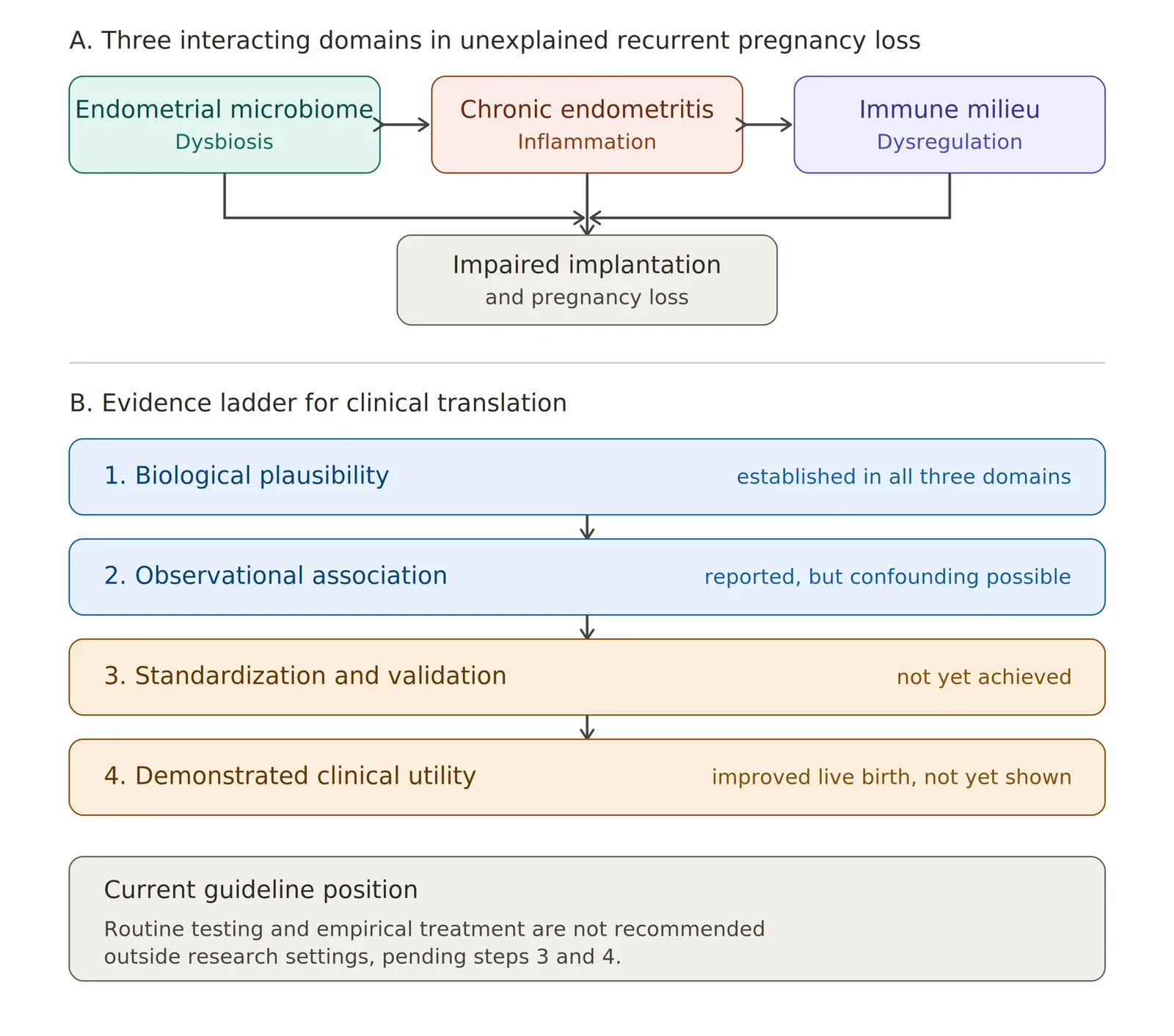
Interacting domains of emerging endometrial biology in unexplained recurrent pregnancy loss and the evidence ladder for clinical translation. (A) The endometrial microbiome (dysbiosis), chronic endometritis (inflammation), and the endometrial immune milieu (dysregulation) are interconnected and may converge on impaired implantation and pregnancy loss. (B) Across all three domains, biological plausibility and observational association are established, but standardization and validation and demonstrated clinical utility (improved live birth) have not yet been achieved. Current guidelines therefore do not recommend routine testing or empirical treatment outside research settings.

The endometrial microbiome, CE, and the endometrial immune milieu are best understood not as three parallel investigational topics but as biologically interconnected components of a shared endometrial environment (Figure 1) [7,8]. Emerging evidence suggests that microbial composition and chronic endometrial inflammation may both influence local immune regulation, and that the three domains may interact in ways that are clinically relevant to implantation and early pregnancy maintenance [9,46]. For example, microbial dysbiosis within the endometrial cavity - if causally implicated - might plausibly promote chronic endometrial inflammation by activating innate immune pattern-recognition pathways, thereby altering the local cytokine milieu and impairing the tolerogenic immune adaptations essential for normal implantation [41,47]. In such a model, CE would represent a histological manifestation of a disturbed host-microbe relationship, and immune dysregulation would represent its functional consequence, so that the three “domains” would be three views of a single underlying process rather than independent risk factors.

These interactions remain largely hypothetical in human studies and are not yet supported by evidence sufficient for clinical application, but they suggest that the field may benefit from more integrative research designs that evaluate multiple domains simultaneously rather than in isolation [8,9,11,46,47]. Prospective cohorts that characterize the microbiome, CE status, and endometrial immune profile in the same patients, using standardized methods and with live birth as the primary outcome, would be far more informative than the current patchwork of single-domain studies.

Across all three fields, the pattern of limitation is strikingly consistent (Table 1). Biological plausibility is well established for each domain. Association with reproductive failure has been reported in multiple observational studies [6]. Yet in each case, the same fundamental barriers recur: methodological heterogeneity between studies, absence of standardized diagnostic criteria or analytical thresholds, inadequate control for confounding variables, predominance of retrospective and observational designs, and insufficient prospective validation in cohorts with RPL as the primary reproductive outcome [11,38,39]. These are not incidental study limitations - they are structural impediments to clinical translation that must be addressed through deliberately designed, adequately powered, multicenter prospective studies.

It is also important for clinicians to recognize that biological plausibility and clinical utility are distinct concepts that require separate demonstration. A biological process may be reproducibly associated with poor reproductive outcomes and yet fail to generate a clinically actionable biomarker or effective treatment - either because the association reflects confounding rather than causation, because diagnostic tests cannot detect the underlying process with adequate reproducibility, or because interventions targeting the process do not deliver the expected clinical benefit. The history of reproductive medicine contains instructive examples of both possibilities: candidate biomarkers with strong biological rationale that failed to demonstrate clinical utility in prospective validation, and, conversely, improved pregnancy outcomes observed in the absence of a clearly validated mechanism. This history counsels caution in translating the current microbiome, CE, and immune findings into practice before the corresponding validation has been completed.

Clinical implications and future directions

The primary implication of the evidence reviewed here is that current guideline-based evaluation should remain the foundation of clinical management for women with unexplained RPL [3-5]. This includes systematic evaluation for established causes - chromosomal, anatomic, immunothrombotic, and endocrine - and the offer of evidence-based interventions where applicable. Unexplained RPL, once established, should be approached with careful counseling that acknowledges the existing biological uncertainty while emphasizing the favorable prognosis that many couples have, particularly those without additional risk factors for poor outcome, given the natural history of spontaneous live birth in subsequent pregnancies [2].

Clinicians will inevitably encounter patients who ask about microbiome testing, CE assessment, or immune evaluation - whether prompted by information encountered online, by other providers, or by personal experience. A clinically useful response to these inquiries should neither dismiss the underlying biological rationale nor overstate the evidence for clinical utility. Clinicians should communicate that these fields are active areas of scientific investigation, that the available evidence does not yet support routine testing or empirical treatment, and that current international guidelines reflect this assessment [28,34]. Where patients express a strong preference for investigation beyond guideline-supported evaluation, shared decision-making should address the genuine uncertainty about clinical benefit, the potential for harm from empirical interventions, and the value of enrolling in research settings where such questions can be rigorously evaluated [48]. Selective testing may be reasonable within such research-oriented or carefully individualized contexts - for example, CE assessment in specific subgroups - provided that both clinician and patient understand its investigational status [35,49]. Microbiome-directed interventions likewise remain investigational: aside from probiotic and vaginal microbiota transplant approaches, the only high-quality randomized evidence for restoring a *Lactobacillus*-dominant state comes from the treatment of bacterial vaginosis rather than from RPL populations, underscoring how far these strategies remain from validated use in RPL [50].

Future research priorities in this field should focus on standardization rather than proliferation of biomarkers. The development of consensus-based, reproducible diagnostic criteria for CE - including harmonization of CD138 immunohistochemical thresholds and biopsy timing - would substantially improve comparability across studies. For the endometrial microbiome, standardization of low-biomass sampling procedures, rigorous contamination controls, extraction protocols, and sequencing analysis pipelines is a prerequisite for reproducible results [22-24]. For immune biomarkers, transition from peripheral blood measurements to validated endometrial assessments, coupled with harmonized laboratory standards, would strengthen the clinical relevance of future studies [11]. In all three domains, prospective multicenter randomized trials with live birth as the primary outcome are required to establish whether identified abnormalities are causally related to pregnancy loss and whether treatment improves outcomes [8,42]. Until such evidence is available, these domains appropriately remain investigational.

For the practicing clinician, three points follow directly from this appraisal. First, when a patient with unexplained RPL asks about endometrial microbiome, CE, or immune testing, the honest answer is that these are areas of active investigation for which current evidence does not support routine testing or empirical treatment outside research settings. Second, the distinction that matters at the bedside is not whether a biological process is real but whether measuring it changes the management in a way that improves live birth - a threshold none of the three domains has yet met. Third, selective testing may still be reasonable within individualized or research contexts, provided both clinician and patient understand its investigational status. Framing the conversation around these points lets clinicians acknowledge the science without overpromising, and supports shared, realistic decision-making.

## Conclusions

Unexplained RPL should be understood as the current boundary between established clinical knowledge and emerging reproductive biology, rather than as evidence that no biological explanation exists. Research into the endometrial microbiome, CE, and the endometrial immune milieu has substantially enriched the understanding of the endometrial environment relevant to implantation and early pregnancy maintenance. Across all three domains, however, biological plausibility and observational association have consistently outpaced the standardized, prospective, outcome-based evidence needed for clinical use. Translating these insights into routine practice therefore requires rigorous evidence that remains outstanding.

Accordingly, current international guidelines do not endorse routine testing for microbial composition, CE, or immune parameters in women with unexplained RPL, nor empirical interventions targeting these domains outside research settings. This position reflects not a rejection of the underlying biology but an appropriately high evidentiary standard, requiring reproducibility, standardized methodology, independent validation, and demonstrated improvement in live-birth rates. As such evidence accumulates, the category of unexplained RPL will continue to evolve. Clinicians can best serve their patients by grounding practice in current guidelines, communicating transparently about the investigational status of these emerging fields, and supporting the research required to translate biological discovery into clinical benefit.

## References

[1] Hyde KJ, Schust DJ (2015). Genetic considerations in recurrent pregnancy loss. Cold Spring Harb Perspect Med.

[2] Cao C, Bai S, Zhang J, Sun X, Meng A, Chen H (2022). Understanding recurrent pregnancy loss: recent advances on its etiology, clinical diagnosis, and management. Med Rev (2021).

[3] Bender Atik R, Christiansen OB, Elson J (2023). ESHRE guideline: recurrent pregnancy loss: an update in 2022. Hum Reprod Open.

[4] (2026). Recurrent pregnancy loss: a committee opinion. Fertil Steril.

[5] (2025). Recommendations on the management of recurrent pregnancy loss (in Japanese). Loss.

[6] Vomstein K, Krog MC, Wrønding T, Nielsen HS (2024). The microbiome in recurrent pregnancy loss - a scoping review. J Reprod Immunol.

[7] Takimoto K, Yamada H, Shimada S, Fukushi Y, Wada S (2023). Chronic endometritis and uterine endometrium microbiota in recurrent implantation failure and recurrent pregnancy loss. Biomedicines.

[8] Yamada H, Ono Y, Ota H, Kobayashi Y, Fukushi Y, Wada S, Arase H (2026). Anti-β2GPI/HLA-DR antibody, chronic endometritis, and uterine endometrial microbiome in women with recurrent pregnancy loss: a prospective cohort study. Microorganisms.

[9] Amiri A, Kargar R, Hosseini FS, Hemmatzadeh M, Mohammadi H (2025). The interplay between vaginal microbiota and immune system in recurrent pregnancy loss and repeated implantation failure. J Reprod Immunol.

[10] Inversetti A, Zambella E, Guarano A, Dell'Avanzo M, Di Simone N (2023). Endometrial microbiota and immune tolerance in pregnancy. Int J Mol Sci.

[11] Shao M, Zhang Y, Tang H (2026). Multi-omics insights into immune tolerance at the maternal-fetal interface in recurrent pregnancy loss: mechanisms, integration challenges, and translational perspectives. Front Immunol.

[12] Dahdouh EM, Kutteh WH (2021). Genetic testing of products of conception in recurrent pregnancy loss evaluation. Reprod Biomed Online.

[13] Perez-Muñoz ME, Arrieta MC, Ramer-Tait AE, Walter J (2017). A critical assessment of the "sterile womb" and "in utero colonization" hypotheses: implications for research on the pioneer infant microbiome. Microbiome.

[14] Moreno I, Codoñer FM, Vilella F (2016). Evidence that the endometrial microbiota has an effect on implantation success or failure. Am J Obstet Gynecol.

[15] Franasiak JM, Werner MD, Juneau CR (2016). Endometrial microbiome at the time of embryo transfer: next-generation sequencing of the 16S ribosomal subunit. J Assist Reprod Genet.

[16] Kyono K, Hashimoto T, Nagai Y, Sakuraba Y (2018). Analysis of endometrial microbiota by 16S ribosomal RNA gene sequencing among infertile patients: a single-center pilot study. Reprod Med Biol.

[17] Riganelli L, Iebba V, Piccioni M (2020). Structural variations of vaginal and endometrial microbiota: hints on female infertility. Front Cell Infect Microbiol.

[18] Odendaal J, Black N, Bennett PR, Brosens J, Quenby S, MacIntyre DA (2024). The endometrial microbiota and early pregnancy loss. Hum Reprod.

[19] Ravel J, Gajer P, Abdo Z (2011). Vaginal microbiome of reproductive-age women. Proc Natl Acad Sci U S A.

[20] Benner M, Ferwerda G, Joosten I, van der Molen RG (2018). How uterine microbiota might be responsible for a receptive, fertile endometrium. Hum Reprod Update.

[21] Giannella L, Grelloni C, Quintili D (2023). Microbiome changes in pregnancy disorders. Antioxidants (Basel).

[22] Molina NM, Sola-Leyva A, Haahr T, Aghajanova L, Laudanski P, Castilla JA, Altmäe S (2021). Analysing endometrial microbiome: methodological considerations and recommendations for good practice. Hum Reprod.

[23] Salter SJ, Cox MJ, Turek EM (2014). Reagent and laboratory contamination can critically impact sequence-based microbiome analyses. BMC Biol.

[24] Eisenhofer R, Minich JJ, Marotz C, Cooper A, Knight R, Weyrich LS (2019). Contamination in low microbial biomass microbiome studies: issues and recommendations. Trends Microbiol.

[25] Liu Y, Wong KK, Ko EY (2018). Systematic comparison of bacterial colonization of endometrial tissue and fluid samples in recurrent miscarriage patients: implications for future endometrial microbiome studies. Clin Chem.

[26] Toson B, Simon C, Moreno I (2022). The endometrial microbiome and its impact on human conception. Int J Mol Sci.

[27] Garmendia JV, De Sanctis CV, Hajdúch M, De Sanctis JB (2024). Microbiota and recurrent pregnancy loss (RPL); more than a simple connection. Microorganisms.

[28] Yuan X, Gao J, Bajinka O, Feng X (2025). Vaginal microbiome and recurrent pregnancy loss. Infect Immun.

[29] Mori R, Hayakawa T, Hirayama M (2023). Cervicovaginal microbiome in patients with recurrent pregnancy loss. J Reprod Immunol.

[30] Shi Y, Yamada H, Sasagawa Y, Tanimura K, Deguchi M (2022). Uterine endometrium microbiota and pregnancy outcome in women with recurrent pregnancy loss. J Reprod Immunol.

[31] Black N, Henderson I, Quenby S, Odendaal J, MacIntyre DA (2026). Microbiota composition of the female reproductive tract and miscarriage: a systematic review and meta-analysis. NPJ Biofilms Microbiomes.

[32] Dube R, Kar SS (2022). Genital microbiota and outcome of assisted reproductive treatment-a systematic review. Life (Basel).

[33] Hashimoto T, Kyono K (2019). Does dysbiotic endometrium affect blastocyst implantation in IVF patients?. J Assist Reprod Genet.

[34] Gao X, Louwers YV, Laven JS, Schoenmakers S (2024). Clinical relevance of vaginal and endometrial microbiome investigation in women with repeated implantation failure and recurrent pregnancy loss. Int J Mol Sci.

[35] Patki A, Kunjimoideen K, Sawankar S, Tyagi R, Hegde V, Budi J (2025). Expert opinion on the use of probiotics in recurrent pregnancy loss. Cureus.

[36] Kitaya K, Takeuchi T, Mizuta S, Matsubayashi H, Ishikawa T (2018). Endometritis: new time, new concepts. Fertil Steril.

[37] Moreno I, Cicinelli E, Garcia-Grau I (2018). The diagnosis of chronic endometritis in infertile asymptomatic women: a comparative study of histology, microbial cultures, hysteroscopy, and molecular microbiology. Am J Obstet Gynecol.

[38] Yasuo T, Kitaya K (2022). Challenges in clinical diagnosis and management of chronic endometritis. Diagnostics (Basel).

[39] Klimaszyk K, Svarre Nielsen H, Wender-Ozegowska E, Kedzia M (2023). Chronic endometritis - is it time to clarify diagnostic criteria?. Ginekol Pol.

[40] Yan X, Jiao J, Wang X (2025). Inflammatory mechanisms and therapeutic advances in chronic endometritis. Front Immunol.

[41] Vidal A, Kilian AY, Vinayahalingam V, Zagrapan B, Pape J, Karrer T, von Wolff M (2026). The role of endometrial microbiota in the pathogenesis of chronic endometritis: a systematic review and meta-analysis. Biomedicines.

[42] Odendaal J, Black N, Bouliotis G, Guck J, Underwood M, Fisher J, Quenby S (2023). Preconceptual administration of doxycycline in women with recurrent miscarriage and chronic endometritis: protocol for the Chronic Endometritis and Recurrent Miscarriage (CERM) trial, a multicentre, double-blind, placebo-controlled, adaptive randomised trial with an embedded translational substudy. BMJ Open.

[43] Agostinis C, Mangogna A, Bossi F, Ricci G, Kishore U, Bulla R (2019). Uterine immunity and microbiota: a shifting paradigm. Front Immunol.

[44] Ticconi C, Pietropolli A, Di Simone N, Piccione E, Fazleabas A (2019). Endometrial immune dysfunction in recurrent pregnancy loss. Int J Mol Sci.

[45] Wang W, Sung N, Gilman-Sachs A, Kwak-Kim J (2020). T helper (Th) cell profiles in pregnancy and recurrent pregnancy losses: Th1/Th2/Th9/Th17/Th22/Tfh cells. Front Immunol.

[46] Bahia W, Soltani I, Ferchichi S, Almawi WY (2026). Mechanisms and therapeutic implications of microbiome-mediated immune dysregulation in recurrent pregnancy loss and implantation failure. Am J Reprod Immunol.

[47] Mikhalev SA, Kurtser MA, Radzinsky VE, Orazov MR, Beeraka NM, Mikhaleva LM (2025). Exploring the role of lower genital tract microbiota and cervical-endometrial immune metabolome in unknown genesis of recurrent pregnancy loss. Int J Mol Sci.

[48] DeLong K, Bensouda S, Zulfiqar F (2019). Conceptual design of a universal donor screening approach for vaginal microbiota transplant. Front Cell Infect Microbiol.

[49] Wrønding T, Vomstein K, Bosma EF (2023). Antibiotic-free vaginal microbiota transplant with donor engraftment, dysbiosis resolution and live birth after recurrent pregnancy loss: a proof of concept case study. EClinicalMedicine.

[50] Cohen CR, Wierzbicki MR, French AL (2020). Randomized trial of lactin-V to prevent recurrence of bacterial vaginosis. N Engl J Med.

